# Rush Medical College, Chicago, Illinois

**Published:** 1845-08-01

**Authors:** 


*Rush Medical College, Chicago, Illinois.*—The third annual course of Lectures in this institution, will take place with some changes in the organization of the Faculty. G. N. Fitch, M. D. of Indiana, has been appointed to the chair of Institutes and Practice of Medicine, which was made vacant by our declining a re-election. Dr. Fitch formely occupied the chair of Midwifery. John Evans, M. D. of Indiana, has been appointed to the latter chair. We are gratified to learn that there are prospects of an increased class for the next session. The location of this school, the interest felt in it by the citizens of Chicago, and the zeal and ability of its faculty, conspire to render it already a flourishing, and, prospectively, a highly successful institution. It is a common impression among the profession that medical schools are becoming too much multiplied, especially those which may be called, relatively, provincial schools. Personal observation, however, would satisfy anyone that with regard to a school located, for example, at Chicago, the question of its necessity resolves itself into this—Shall a large portion of the present and future practitioners of Illinois, Indiana, Wisconsin, Iowa, &c. have the advantages of a school contiguous to themselves, or of none whatever? In other words, a large portion of the medical students will not go to a distant school to attend medical lectures, and if a medical school does not come to them, or near to them, they commence practitioners without this preliminary. We participated in the impression above stated, until personal observation convinced us that it was erroneous. As to the question whether the multiplication of medical schools tends to useful or injurious results, if required to give an opinion, we should perhaps incline to do so in the manner of Sir Roger de Coverly, by deciding that "much is to be said on both sides." The question is an important one, and unless discussed by some of our correspondents, we may undertake it at some convenient season. We will say, however, here, that we are by no means disposed to attach to the number, or even to the character of medical schools the influences which some appear to think flow from this source. The over-crowded state of the profession, and the low standard of medical education, are probably, due to other causes—causes with which medical schools are not identified, but in the effects of which they participate, in common with every department of the profession.

